# Preliminary Evaluation of Thulium Doped Fiber Laser in Pig Model of Liver Surgery

**DOI:** 10.1155/2018/3275284

**Published:** 2018-10-15

**Authors:** Maciej Janeczek, Jacek Świderski, Albert Czerski, Bogusława Żywicka, Jolanta Bujok, Maria Szymonowicz, Ewa Bilewicz, Maciej Dobrzyński, Mariusz Korczyński, Aleksander Chrószcz, Zbigniew Rybak

**Affiliations:** ^1^Department of Animal Physiology and Biostructure, Division of Anatomy, Wroclaw University of Environmental and Life Sciences, Kożuchowska 1, 51-631 Wroclaw, Poland; ^2^Institute of Optoelectronics, Military University of Technology, Kaliskiego 2, 00-908 Warsaw, Poland; ^3^Department of Animal Physiology and Biostructure, Division of Animal Physiology, Wroclaw University of Environmental and Life Sciences, C.K. Norwida 31, 50-375 Wroclaw, Poland; ^4^Department of Experimental Surgery and Biomaterial Research, Wroclaw Medical University, Bujwida Street 44, 50-368 Wroclaw, Poland; ^5^Department of Conservative Dentistry and Pedodontics, Wroclaw Medical University, Krakowska 26, 50-425 Wroclaw, Poland; ^6^Department of Environment Hygiene and Animal Welfare, Wroclaw University of Environmental and Life Sciences, Chelmonskiego 38c, 51-630 Wroclaw, Poland

## Abstract

Partial liver resection is a treatment of choice for liver tumors; the range of parenchyma excision varies from a small part of the tissue surrounding the neoplasm up to 70% of the organ. One of the major concerns during liver resection is blood loss. Thulium lasers which are characterized by the length of emission wave corresponding to a peak absorption of water create a new possibility of cutting tissues efficiently with minimal thermal damage and concurrently providing a good hemostasis control. The aim of our study was to evaluate an impact of liver transection with thulium doped fiber laser on an intraoperative bleeding and histopathological changes during postoperative period in swine model. Ten animals were subjected to open surgery partial liver resection and an incision of liver tissue with an all-fiber, diode-pumped, and continuous-wave Tm^3+^-doped fiber laser emitting 37.4 W of output power at ~1.94 *μ*m wavelength. The macroscopic and histopathological evaluation was performed intraoperatively as well as 7 and 14 days after surgery. Macroscopically almost no bleeding was observed during surgery and no signs of bleeding were stated after 7 and 14 days. Histopathological analysis of the transection margin revealed a thermal damage area ranging in depth from 620.23 ± 23.82 *μ*m on the day of surgery to 1817.70 ± 211.98 *μ*m after 7 days. In the samples taken intraoperatively and after 7 days a superficial zone of carbonization was visibly separated from the deeper changes. After 14 days one 765.35* μ*m deep zone characterized by a granulation was present. In conclusion, thulium doped fiber laser is efficacious in cutting with a narrow zone of thermal injury and provides a good hemostasis during liver transection, thus being a potential tool for oncotic liver surgery.

## 1. Introduction

Partial liver resection is the standard treatment for primary and secondary liver tumors. The two most common indications for liver resection are colorectal liver metastases and hepatocellular carcinomas, and the range of liver parenchyma resection varies from limited to the tissue surrounding the tumor to up to 70% of the organ [1, 2]. Although the liver resection technique is being constantly improved and the perioperative complication rate markedly decreased, it still remains a demanding surgical procedure with significant mortality and morbidity [3]. One of the major concerns during liver surgery is blood loss, as it has been demonstrated to be one of the main predictors of perioperative complications and death [4]. Many novel interventional techniques are aimed at reducing blood loss during liver parenchyma transection and managing the cut surface [1]. Another objective is to perform liver tumor resections with safe tissue margins, while leaving as much tissue as possible, especially if the patient is suffering from liver cirrhosis. Excessive excision of the liver parenchyma in this case may result in an organ failure. Therefore, a device allowing great hemostatic control and precise cut with maximum sparing of the unaffected tissue would be of advantage in liver surgery.

Soon after Maiman in 1960 discovered the first ruby-based working laser, many types of gaseous, liquid, and solid materials were found applicable in a laser technique [5]. Concurrently, first experiments on medical laser application begun and only a couple of years after Maiman's discovery, ruby laser was studied in ophthalmology leading to an extensive use of modern laser devices in this branch of medicine [6, 7]. Currently, medical laser devices are widely used not only in ophthalmology, but also in laryngology, dermatology, urology, oncology, and surgery, and the number of procedures performed with lasers continually grows. Several types of lasers are used in soft tissue surgery, inter alia carbon dioxide (CO_2_), neodymium-doped yttrium aluminum garnet (Nd:YAG), and diode lasers [8–12]. Thulium doped fiber (Tm:fiber) lasers with the wavelength close to 2000 nm which corresponds to peak absorption of water are potentially promising tools in surgery, characterized by high efficiency and minimal thermal damage of the surrounding tissues [13]. However, data on its applicability in liver surgery are sparse.

The aim of our study was to evaluate an impact of liver transection with all-fiber, diode-pumped, continuous-wave Tm^3+^-doped fiber laser on an intraoperative bleeding and histopathological changes during postoperative period in swine model.

## 2. Materials and Methods

### 2.1. Study Protocol and Animals

The experiment was approved by the Second Wroclaw Local Ethics Committee for Animal Experimentation (no. 50/2012) and performed in accordance with the standards established in the directive of the EU (2010/63/EU). 10 Polish Large White (WBP) female pigs aged 10 weeks were used in this study. The animals were obtained from a single farm (The National Research Institute of Animal Production, Experimental Station in Pawłowice, Poland) and acclimatized for 2 weeks before enrolling into one of two equal groups. In both experimental groups, the animals underwent partial hepatectomy of the right lateral lobe and incision of the liver parenchyma under general anesthesia. The excised fragments of tissue were assessed by histopathology (HP). After recovery from anesthesia, pigs were kept in the pens of a vivarium for 7 days (T1 group) or for 14 days (T2 group) and, after this period of time, were euthanized with intravenous pentobarbitone (48–96 mg/kg; Morbital®, Biowet Puławy, Poland) to obtain tissues for HP.

### 2.2. Laser Properties

The thulium laser used in the experiment was a prototype all-fiber, diode-pumped, and continuous-wave Tm^3+^-doped fiber laser. The laser was built with the use of a mode area single-mode thulium doped fiber. The silica core fiber 25 *μ*m in diameter (0.09 NA) placed in a 250 *μ*m octagonal clad fiber (0.46 NA) was pumped by one 793 nm laser diode with 200 *μ*m diameter (0.22 NA) pigtail fiber. Optical cavity of the laser consisted of two fiber Bragg gratings with the wavelength of 1940 nm; the first was highly reflective and the second partially reflective and served as an output coupler fused with a passive transmitting fiber which ended with a high power FC/APC connector. A dichroic mirror at the output end was filtering the residual diode pump light and 1940 nm laser light. Tm:fiber laser was emitting 37.4 W of output power at ~1.94 *μ*m wavelength. The device used in this study was constructed in the Institute of Optoelectronics, Military University of Technology (Warsaw, Poland), and its properties were described in detail by Michalska et al. [14].

### 2.3. Surgery and Visual Inspection

The animals were sedated with intramuscular (i.m.) injection of medetomidine (0.1 mg/kg body weight; Domitor®, Orion Pharma, Poland) and butorphanol (0.2 mg/kg body weight; Butomidor®, Richter Pharma AG, Austria) and were inserted a venous catheters into the auricular veins. Intravenous (i.v.) propofol bolus (4 mg/ kg; Scanofol®, Scan Vet, Poland) was used to induce anesthesia in pigs, to allow their placement in the dorsal recumbent position and tracheal intubation. General anesthesia was maintained with 1.5%vol isoflurane. Analgesia was provided with an i.v. constant-rate infusion of fentanyl (50 *μ*g/kg/10 min, Polfa SA, Poland). Each animal received 500 ml of Ringeri solution i.v. during the procedure. Surgical access was achieved by a midline incision through the linea alba. The right lateral lobe of the liver was identified visually and gently fixed with a sterile gauze. A partial incision and hepatectomy were performed in the 2/3 distal part of the lobe, where the thickness of the tissue was approximately 2 cm. The tip of the laser probe during cutting was kept at a distance of 2-5 mm from the tissue surface. The cut area was observed for 5 min to visualize bleeding or bile leakage. Afterwards, the abdominal cavity was closed in three layers. Before weaning form anesthesia the animals were given amoxicillinum (15 mg/kg body weight i.m.; Betamox LA®, ScanVet, Poland) and metamizole (30 mg/kg body weight i.m.; Biovetalgin®, Biowet Drwalew, Poland) and placed in the pens. In the remaining period of the experiment (7 days and 14 days for T1 and T2 group, respectively) animals were observed for any signs of pain and their clinical status was evaluated. Pain was controlled with metamizole i.m. for 3 days after surgery.

### 2.4. Macroscopic Evaluation and Histopathology

After euthanasia, pigs were subjected to necropsy with a particular emphasis on the signs of bleeding or bile leakage from the site of resection, and the specimens from this part of the liver were collected for HP.

Specimens obtained either during surgery or necropsy were immediately fixed in 4% buffered formaldehyde for 72 h and then washed in running water for 24 h. Before embedding in paraffin (Microm EC 350-1, Thermo Scientific, USA), specimens were dehydrated successively in 75%, 96%, and 100% solutions of ethanol. Paraffin blocks with the liver samples were cut into 7 *μ*m sections using a sliding microtome (Zeiss Hyrax M25, Carl Zeiss Company, Germany) and were placed on glass slides. The sections were stained with hematoxylin and eosin (HE; Sigma-Aldrich, USA) according to the standard protocols [15]. Stained specimens were evaluated under the light microscope (Zeiss Axio Scope A1, Carl Zeiss Company, Germany). Histological measurements were made using Axio Vision 4.8 (Carl Zeiss MicroImaging GmbH, Germany).

### 2.5. Statistics

Data are expressed as mean and standard deviation of average values from measurements made in specimens obtained from n animals. Data were subjected to Student's* t*-test analysis using the Statistica for Windows ver. 10.0 software package (StatSoft, Tulsa, OK, USA). Differences between means were considered significant at values of p < 0.05.

## 3. Results

### 3.1. Animals and Macroscopic Evaluation

All animals survived the surgical procedure without any complications. They remained in good clinical condition and had a good appetite until the end of the experiment and were euthanized after 7 or 14 days in T1 or T2 group, respectively.

**Figure 1:**
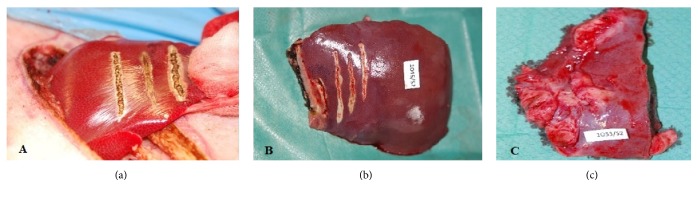
Macroscopic evaluation of right lateral liver lobe after thulium laser surgery. (a) Incision of the liver tissue with a thulium laser (first from the left), image taken intraoperatively; (b) macroscopic changes in pig liver on day 7; carbonization is visible in the resection wound; incomplete healing of the wound after incision (the first from the right); (c) a scar formation in the liver on day 14 (T2 group) after partial resection.

Intraoperatively not any signs or minor signs of bleeding during 5 minutes after cutting the tissue with Tm:fiber laser were observed in the pigs from T1 and T2 groups. No bile leakage was observed. On the surface of the excision and incision margins a carbonization was present, which macroscopically formed a 1-2 mm deep zone in the liver lobe. After 7 days carbonization was still visible, while after 14 days only small foci of carbonization remained. No extravasated blood, clots, or any other signs suggesting delayed bleeding or bile leakage were present in T1 and T2 group. After 14 days adhesion of the liver with abdominal wall or neighboring organs at the site of cutting was visible. Tissue of the liver lobe apart from the incision line showed no macroscopic changes in any of the animals (Figure 1).

### 3.2. Histopathology

**Figure 2:**
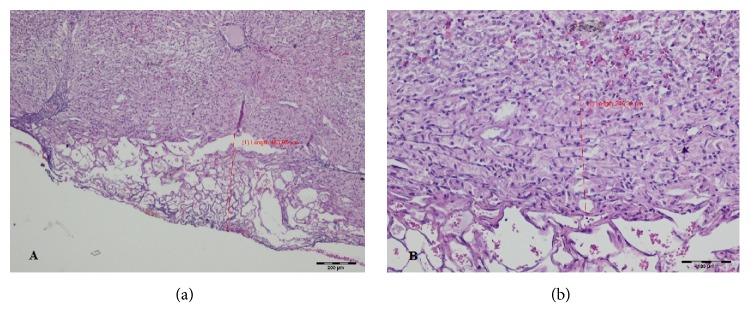
Histopathological evaluation of the fragment of the liver excised with thulium laser on day 0 (HE staining). (a) Magnification ×40; (b) magnification×100.

**Figure 3:**
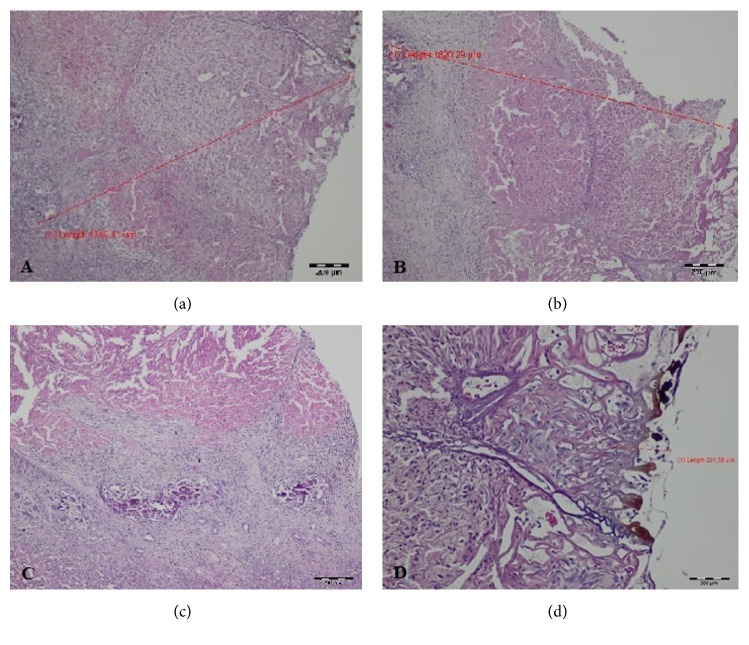
Histopathological evaluation of the liver tissue damage on day 7 after cutting with thulium laser (magnification x40, HE staining). (a) and (b) Total depth of thermal tissue damage; (c) focal necrotic lesions; (d) clear zone of carbonization in the middle of the incision wound.

**Figure 4:**
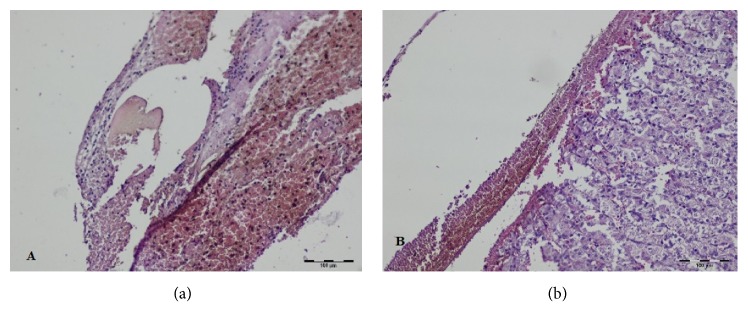
Histopathological evaluation of the liver tissue at the site of transection with thulium laser on day 14 ((a) and (b) magnification x100, HE staining).

During HP evaluation of specimens taken intraoperatively and 7 days after surgery the superficial and deep zones of changed tissue were distinguished, whereas in T2 group both zones merged. The width of the thermally changed area and its microscopic appearance varied with time (Table 1, Figures 2–4).

The superficial zone of the microscopic changes in specimens collected intraoperatively was characterized by exudation and carbonization without extravasated erythrocytes. In the deeper zone of thermal damage morphologically altered lobules were present; however, hepatic cells themselves had a preserved structure and intact nuclei. This layer was clearly separated from the normal liver parenchyma (Figure 2).

In the samples from T1 group a superficial zone with carbonized tissue and exudative phase was still present; however, the width of this layer decreased significantly (Table 1). The deeper zone was characterized by the granulation tissue area with large number of small blood vessels with thin walls. In this area the proliferation phase with mesenchymal cells, hepatocytes, and fibroblasts was present. Deeper in this zone of thermal damage the necrotic foci surrounded by the inflammatory cells were seen. The width of the changes in the liver tissue produced by the Tm:fiber laser was the largest 7 days after surgery (Figure 3).

In the samples taken 14 days after surgery both zones merged and formed a layer of isolated areas of laser activity filled with the granulation tissue and the residues of carbonized tissue. These areas were surrounded by granulocytes mostly neutrophils, by macrophages, lymphocytes, plasmatic cells, and mesenchymal cells. In T2 group the total width of changed tissue decreased comparing with samples taken 7 days after surgery (Figure 4).

## 4. Discussion

Liver resection remains a surgery not without complications, of which a blood loss is the most serious concern. Different methods of vascular occlusion are applied, and new techniques and devices for parenchymal transection are sought to minimize bleeding. Among others cavitron ultrasonic surgical aspirator, ultrasonic shears, and radiofrequency dissecting sealer are the devices designed to cut liver parenchyma with minimal bleeding [16].

In the middle 80s of XX century different types of lasers were tested for their feasibility in liver surgery. Most attention was turned to Nd:YAG lasers and CO_2_ lasers. Na:YAG laser was compared with ultrasonic surgical aspirator and blunt dissection in a dog model of liver resection. It was not superior to the other two techniques in particular due to a large zone of thermal damage [17]. In a pig model of liver resection Nd:YAG laser, CO_2_ laser and a combination of these two were tested. CO_2_ laser had a better cutting efficiency and produced a narrow thermal damage zone approximately 1.5 mm wide, but it did not provide hemostasis. Na:YAG laser was characterized by a good hemostatic control; however, it produced a broad zone of thermal damage in the cutting margin, from 2.4 to 5.8 mm depending on the mode of the laser and power used [18]. Although the combination of both lasers gave better effects, neither of these types of lasers was superior to nonlaser techniques of hepatic parenchyma transection, and thus the laser technique had not been transposed to a clinical practice. Later two Nd:YAG lasers (1064 and 1318 nm) were studied in a porcine model of laparoscopic liver surgery. The use of 60 W output energy provided a coagulation of blood vessels up to 2 mm in diameter and produced a total lateral damage zone of 4.0 mm, what was comparable with earlier studies [19, 20]. In ex vivo studies a 120 W Nd:YAG laser produced in pig liver parenchyma a coagulation zone of approximately 255.9  ±  1.6 to 375.6  ±  2.3 *μ*m, which is comparable to our results in specimens taken intraoperatively. However, the different experimental conditions, inter alia the lack of organ perfusion in ex vivo model may be a limitation in a direct comparison of these two types of lasers [21]. Currently, Nd:YAG lasers have another application in oncotic liver surgery, namely, laser-induced thermotherapy (LITT). In LITT, a low power laser energy produces a well-defined area of coagulative necrosis, thus enabling neoplastic cells damage in situ. Nd:YAG laser with diffusing applicator due to its wavelength of 1046 nm penetrates the surrounding tissue producing immediate and delayed thermal damage, which makes it feasible for treatment of small tumors not suitable for surgical resection [22].

Thulium doped fiber lasers are promising devices in surgery due to several favorable features. Firstly, they operate at a wavelength of approximately 1940-2000 nm, which corresponds to the length of light highly absorbed by water. Most of the soft tissues is characterized by an abundance of water, and similarly, porcine liver has been shown to have a maximum absorption coefficient at 1940 nm [23]. This Tm:fiber laser property results in a shallow penetration of the laser energy into the tissue, followed by a strong local thermal effect without damaging the deeper structures. Moreover, it provides a better cutting precision. In our study Tm:fiber laser had very good cutting properties while the total depth of thermal changes in the liver tissue was from 0.62 to 1.82 mm depending on the time of evaluation. Similar width of the thermal damage zone (less than 2.0 mm) was reported in an experimental pig model of partial nephrectomy with Tm:fiber laser. In another pig model of atypical liver resection with Tm:fiber laser emitting a wavelength of ~ 1.9* μ*m in a contact mode a narrow scar width of approximately 1-2 mm after 14-16 days was also noted [24, 25]. However, in a pig model of laparoscopic liver resection 11-15 days after surgery the total thermal damage zone exceeded 5 mm [26]. The differences might be attributed to the higher power used and other operating techniques. Moreover, in this model a time course of histopathological changes and the initial depth of tissue damage were not studied. Laser energy may cause a direct ultrastructural damage subsequently leading to cell death, what manifested as the largest zone of thermal damage 7 days after surgery in our study. Moreover, thermal ablation of prostate with a Tm:fiber laser emitting at 1940-nm in an in vitro model utilizing dog tissues resulted in a 0.5 to 2.0 mm zone of thermal coagulation, whereas when cutting the bladder wall and ureter the margins of thermal changes did not exceed 1 mm [27]. A very precise cutting and a narrow margin of thermal damage achieved with Tm:fiber laser allow sparing more healthy tissue, what may be of advantage in oncologic liver surgery.

Another advantageous feature of the Tm:fiber laser is that the potent local thermal effect resulting in its ability to cut is accompanied by coagulation, thus providing hemostasis. Control of blood loss is a common problem in surgery of highly vascularized tissues as kidney, spleen, or liver. As opposed to early studies on CO_2_ laser in liver resection, in which an efficient cutting did not produce hemostasis [18], we proved that Tm:fiber laser is able to provide a good bleeding control during hepatic transection. In our experiment no visible blood loss or very minor bleeding was observed after resection of distal 2/3 of right lateral lobe in swine. Similar results were observed by Theisen-Knude et al. [25] in porcine model of partial liver resection with a 1.9 *μ*m thulium laser. An atypical partial hepatectomy by open and laparoscopic techniques did not result in significant bleeding or bile leakage, suggesting feasibility of the Tm:fiber laser in liver surgery [25, 26]. Moreover, in experimental partial nephrectomy using Tm:fiber laser, good hemostasis was observed [24]. Thulium laser with a wavelength of 2 *μ*m has been used for partial nephrectomy in five human patients as well, the control of blood loss was very good, and authors discussed a possibility of performing the procedure without hilar clamping [28]. This thesis was further supported by performing the experimental partial nephrectomy without hilar control conducted in the pig model with thulium YAG laser as well as in human patients undergoing partial nephrectomy for kidney tumors [29, 30]. Moreover, thulium laser technique for prostate enucleation was proved to be an efficient procedure especially in patients with high prostate volumes. It also minimizes blood loss and might be considered the best choice for patients under concurrent antiplatelet therapy [31, 32].

## 5. Conclusion

In conclusion Th:fiber laser is efficacious in cutting with a narrow zone of thermal injury and provides good hemostasis during partial liver resection and liver tissue incision. Tm:fiber laser operating at 1940 nm may be a potential tool in oncologic liver surgery, especially when sparing of a healthy tissue is being a priority and small atypical excisions are performed.

## Figures and Tables

**Table 1:** Width of the microscopic changes in the right lateral lobe of the swine liver produced by the partial resection using a Tm:fiber laser.

Specimen	T1 and T2 intraoperatively (n=10)	T1 (n = 5)	T2 (n = 5)
Zone of superficial thermal damage in the liver tissue [*μ*m]	479.31 ± 13.15	291.60 ± 18.28^*∗*^	-

Total width of microscopic thermal changes in the liver tissue [*μ*m]	620.23 ± 23.82	1817.70 ± 211.98^*∗*^	765.35 ± 55.94

T1 and T2: groups of animals undergoing euthanasia on days 7 and 14, respectively. Values are presented as mean ± SD; n: number of animals. Values marked with *∗* differ significantly (p<0.05) from others in a row.

## Data Availability

The data used to support the findings of this study are included within the article.
